# Aripiprazole and Haloperidol Activate GSK3β-Dependent Signalling Pathway Differentially in Various Brain Regions of Rats

**DOI:** 10.3390/ijms17040459

**Published:** 2016-03-28

**Authors:** Bo Pan, Xu-Feng Huang, Chao Deng

**Affiliations:** 1 Illawarra Health and Medical Research Institute, Wollongong 2522, Australia; bp355@uowmail.edu.au (B.P.); xhuang@uow.edu.au (X.-F.H.); 2 School of Medicine, University of Wollongong, Wollongong 2522, Australia

**Keywords:** antipsychotics, aripiprazole, β-catenin, bifeprunox, Dvl-3, GSK3β, haloperidol

## Abstract

Aripiprazole, a dopamine D_2_ receptor (D_2_R) partial agonist, possesses a unique clinical profile. Glycogen synthase kinase 3β (GSK3β)-dependent signalling pathways have been implicated in the pathophysiology of schizophrenia and antipsychotic drug actions. The present study examined whether aripiprazole differentially affects the GSK3β-dependent signalling pathways in the prefrontal cortex (PFC), nucleus accumbens (NAc), and caudate putamen (CPu), in comparison with haloperidol (a D_2_R antagonist) and bifeprunox (a D_2_R partial agonist). Rats were orally administrated aripiprazole (0.75 mg/kg), bifeprunox (0.8 mg/kg), haloperidol (0.1 mg/kg) or vehicle three times per day for one week. The levels of protein kinase B (Akt), *p*-Akt, GSK3β, p-GSK3β, dishevelled (Dvl)-3, and β-catenin were measured by Western Blots. Aripiprazole increased GSK3β phosphorylation in the PFC and NAc, respectively, while haloperidol elevated it in the NAc only. However, Akt activity was not changed by any of these drugs. Additionally, both aripiprazole and haloperidol, but not bifeprunox, increased the expression of Dvl-3 and β-catenin in the NAc. The present study suggests that activation of GSK3β phosphorylation in the PFC and NAc may be involved in the clinical profile of aripiprazole; additionally, aripiprazole can increase GSK3β phosphorylation via the Dvl-GSK3β-β-catenin signalling pathway in the NAc, probably due to its relatively low intrinsic activity at D_2_Rs.

## 1. Introduction

Aripiprazole is an atypical antipsychotic drug with therapeutic effects on both positive and negative symptoms of schizophrenia, but reduced extrapyramidal side-effects (EPS) compared with typical antipsychotics (e.g., haloperidol) [1]. The exact mechanisms of aripiprazole remain unclear. Glycogen synthase kinase 3β (GSK3β) has been implicated in the pathophysiology of schizophrenia and the actions of antipsychotic drugs [2]. GSK3β is a major downstream regulator of dopamine D_2_ receptors (D_2_Rs), which is targeted by most antipsychotics (including aripiprazole) [3]. Activation of D_2_Rs facilitates the formation of the β-arrestin2-protein phosphatase 2A-protein kinase B (PKB or Akt) complex, resulting in dephosphorylation of Akt (inactivation), followed by dephosphorylation (activation) of GSK3β [4,5,6]. Aripiprazole has been shown to have effects on regulating the Akt-GSK3β signalling pathway [2]. For example, Seo *et al.* [7] have revealed that aripiprazole altered GSK3β activity in the frontal cortex. However, whether aripiprazole can affect GSK3β activity in other schizophrenia-related brain regions has not yet been studied. Our previous acute study [8] has found that acute administration of aripiprazole increased the phosphorylation levels of GSK3β in various brain regions, including the prefrontal cortex (PFC), caudate putamen (CPu), and nucleus accumbens (NAc). However, it is interesting that Akt did not show parallel changes with GSK3β after acute administration [8]. One possibility is that aripiprazole might affect GSK3β activity via alternative pathway(s) that is independent of Akt. One candidate pathway is the dishevelled (Dvl)-GSK3β-β-catenin signalling pathway. *In vitro* evidence has suggested that various antipsychotics (e.g., clozapine, haloperidol) increase the cellular levels of Dvl and β-catenin via affecting D_2_Rs [9,10]. *In vivo* studies have reported that antipsychotic drug administration (including aripiprazole and haloperidol) promoted phosphorylation of GSK3β and expression of Dvl and β-catenin in various brain regions [10,11,12,13,14]. It has been also revealed that administration of aripiprazole attenuated the decreased phosphorylation of GSK3β and reduced expression of β-catenin in the frontal cortex and hippocampus caused by immobilisation stress [7,15]. It should be noted that all these previous studies used intramuscular or subcutaneous injections to deliver aripiprazole. The effects of oral administration that mimic the clinical situation is of importance. Therefore, in this study we examined the Dvl-GSK3β-β-catenin signalling pathway after sub-chronic oral administration of aripiprazole.

Aripiprazole is a D_2_R partial agonist. Researchers have attributed the unique clinical profile of aripiprazole to its partial agonism at D_2_Rs [16,17]. However, the role that D_2_R partial agonism plays in the regulation of the Dvl-GSK3β-β-catenin signalling pathway by aripiprazole is not clear. To investigate this issue, we chose a potent D_2_R partial agonist—bifeprunox [18] to compare with aripiprazole. Therefore, the present study examined the different effects of one-week oral administration of aripiprazole on the Akt-GSK3β and Dvl-GSK3β-β-catenin signalling pathways in three schizophrenia-related brain regions in comparison with a D_2_R antagonist—haloperidol and a D_2_R partial agonist—bifeprunox.

## 2. Results

### 2.1. Effects of Antipsychotics in the Prefrontal Cortex

**Figure 1:**
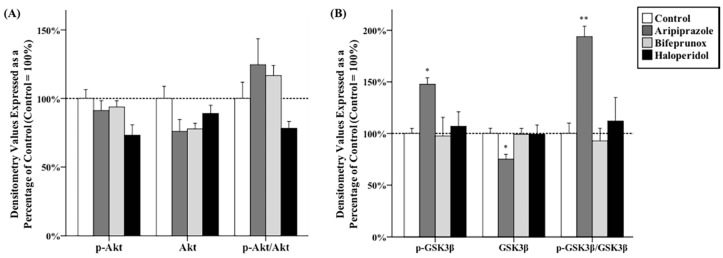

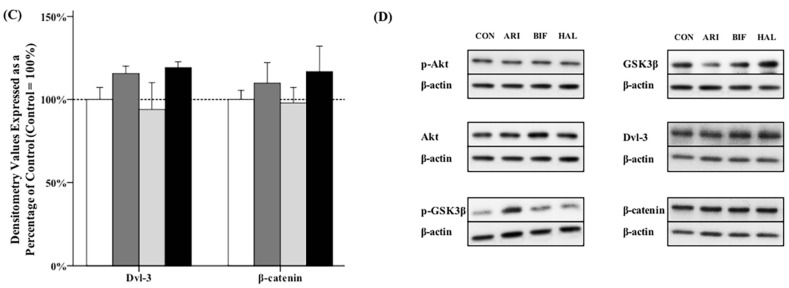
Effects of three antipsychotics in the prefrontal cortex. The effects of aripiprazole (ARI), bifeprunox (BIF), haloperidol (HAL), and control (CON) on the activity of protein kinase B (Akt) (**A**); glycogen synthase kinase 3β (GSK3β) (**B**); and the expression of dishevelled (Dvl)-3 and β-catenin (**C**) were measured in the prefrontal cortex (* *p* ≤ 0.05, ** *p* < 0.01 *vs.* the control). All data were expressed as mean ± S.E.M. The representative bands of Western blot are shown in (**D**).

Antipsychotic drug administration had significant effects on the expression of total GSK3β (*F*_3,20_ = 3.656, *p* < 0.05), p-GSK3β (*F*_3,20_ = 3.722, *p* < 0.05) and the ratio of p-GSK3β/GSK3β (*F*_3,20_ = 9.207, *p* < 0.01) in the PFC, but had no effect on Akt, *p*-Akt, or the ratio of *p*-Akt/Akt (Figure 1A,D). *Post hoc* tests demonstrated that administration of aripiprazole significantly increased the protein levels of p-GSK3β by 47.7% ± 6.4% (*p* < 0.05), but reduced total GSK3β expression by 24.9% ± 4.7% (*p* < 0.05) compared with the control; the ratio of p-GSK3β/GSK3β was also increased by administration of aripiprazole (*p* < 0.01) (Figure 1B,D). Furthermore, the protein levels of Dvl-3 and β-catenin in the PFC were not significantly altered by any antipsychotic drug administration (Figure 1C,D).

### 2.2. Effects of Antipsychotics in the Caudate Putamen

**Figure 2:**
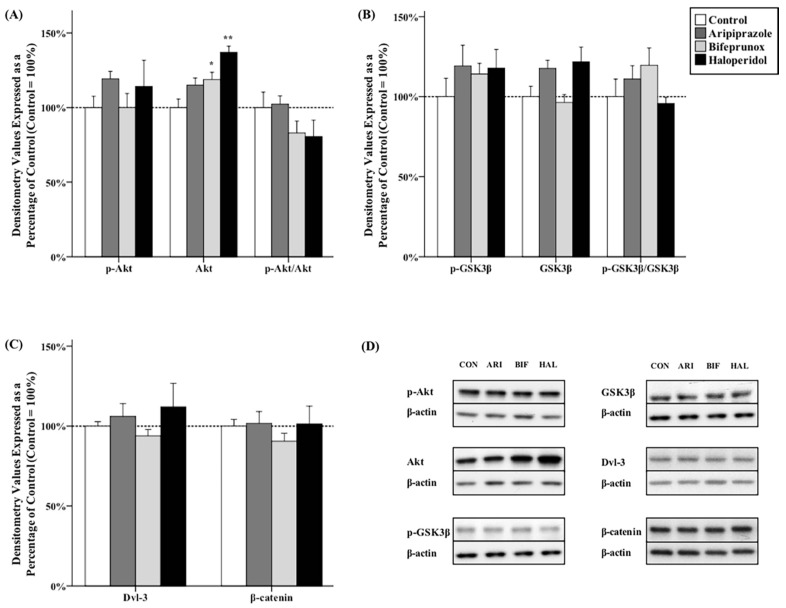
Effects of three antipsychotics in the caudate putamen. The effects of aripiprazole (ARI), bifeprunox (BIF), haloperidol (HAL), and control (CON) on the activity of Akt (**A**); GSK3β (**B**); and the expression of Dvl-3 and β-catenin (**C**) were measured in the caudate putamen (* *p* ≤ 0.05, ** *p* < 0.01 *vs.* the control). All data were expressed as mean ± S.E.M. The representative bands of Western blot are shown in (**D**).

One-way analysis of variance (ANOVA) tests indicated significant effects of antipsychotics on the protein levels of total Akt (*F*_3,20_ = 9.707, *p* < 0.01) in the CPu. *Post hoc* tests showed that the levels of total Akt were significantly increased by administration of bifeprunox (+18.7% ± 4.8%, *p* < 0.05) and haloperidol (+37.0% ± 4.0%, *p* < 0.01) in the CPu (Figure 2A,D); however, they did not affect the levels of *p*-Akt, nor the ratio of *p*-Akt/Akt. Additionally, the protein levels of Dvl-3 and β-catenin were not significantly affected by any antipsychotic drug administration in the CPu (Figure 2B–D).

### 2.3. Effects of Antipsychotics in the Nucleus Accumbens

**Figure 3:**
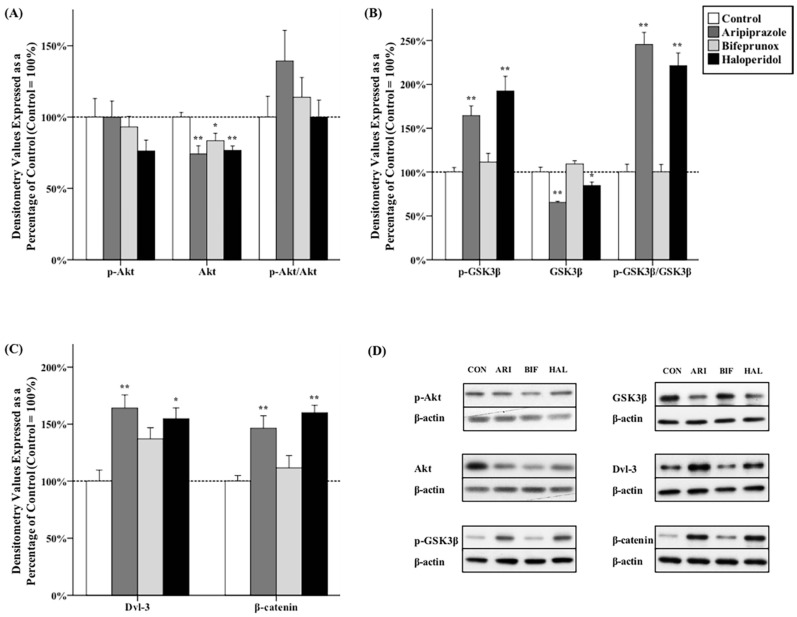
Effects of three antipsychotics in the nucleus accumbens. The effects of aripiprazole (ARI), bifeprunox (BIF), haloperidol (HAL), and control (CON) on the activity of Akt (**A**); GSK3β (**B**); and the expression of Dvl-3 and β-catenin (**C**) were measured in the nucleus accumbens (* *p* ≤ 0.05, ** *p* < 0.01 *vs.* the control). All data were expressed as mean ± S.E.M. The representative bands of Western blot are shown in (**D**).

**Figure 4:**
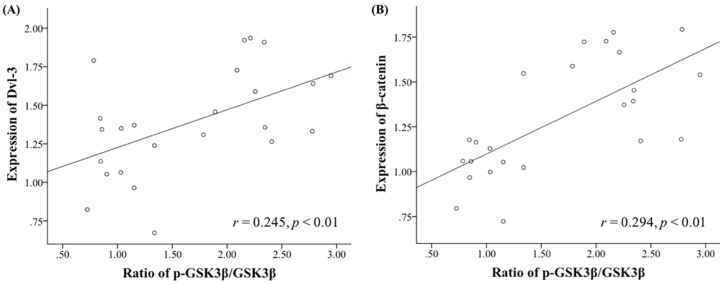
Correlations between the ratio of p-GSK3β/GSK3β and the expression of Dvl-3 and β-catenin in the NAc. The ratio of p-GSK3β/GSK3β is positively correlated with the expression of Dvl-3 (**A**); and with the expression of β-catenin in the NAc (**B**).

ANOVA tests revealed that antipsychotic drug administration had significant effects on the protein levels of Akt (*F*_3,20_ = 6.792, *p* < 0.01), GSK3β (*F*_3,20_ = 25.381, *p* < 0.01), p-GSK3β (*F*_3,20_ = 11.817, *p* < 0.01), the ratio of p-GSK3β/GSK3β (*F*_3,20_ = 42.603, *p* < 0.01), Dvl-3 (*F*_3,20_ = 4.121, *p* < 0.01), and β-catenin (*F*_3,20_ = 10.718, *p* < 0.01) in the NAc. *Post hoc* tests indicated that administration of all three chemicals was shown to be able to reduce the protein levels of total Akt (aripiprazole, −25.9% ± 5.9%, *p* < 0.01; bifeprunox, −16.5% ± 5.0%, *p* < 0.05; haloperidol, −23.4% ± 3.2%, *p* < 0.01) in the NAc; however, no antipsychotic drug administration significantly affected the protein levels of *p*-Akt, nor the ratios of *p*-Akt/Akt (Figure 3A,D). Additionally, the expression of total GSK3β was reduced by both aripiprazole and haloperidol administration (aripiprazole, −34.5% ± 1.2%, *p* < 0.01; haloperidol, −15.3% ± 7.8%, *p* < 0.05). Moreover, both aripiprazole and haloperidol administration was able to elevate the levels of p-GSK3β (aripiprazole, +64.4% ± 11.0%, *p* < 0.05; haloperidol, +92.4% ± 16.7%, *p* < 0.01) and the ratios of p-GSK3β/GSK3β (aripiprazole, *p* < 0.01; haloperidol, *p* < 0.01) (Figure 3B,D). Furthermore, it was shown that administration of aripiprazole was able to promote the expression of both Dvl-3 (+64.1% ± 11.5%, *p* < 0.01) and β-catenin (+46.5% ± 10.7%, *p* < 0.01); haloperidol administration also had a positive effect on the protein levels of both Dvl-3 (+54.8% ± 9.4%, *p* < 0.05) and β-catenin (+59.9% ± 6.6%, *p* < 0.01) (Figure 3C,D). Lastly, we found that the ratio of p-GSK3β/GSK3β is positively correlated with the expression of Dvl-3 in the NAc (*r* = 0.245, *p* < 0.01) (Figure 4A); the ratio of p-GSK3β/GSK3β is also positively correlated with the expression of β-catenin (*r* = 0.294, *p* < 0.01) (Figure 4B).

## 3. Discussion

The present study has examined the effects of aripiprazole on the Akt-GSK3β and Dvl-GSK3β-β-catenin signalling pathways in three key brain regions that are related to the pathophysiology of schizophrenia, in comparison with bifeprunox and haloperidol. Our findings have provided *in vivo* evidence that aripiprazole is able to alter the activity of GSK3β in the PFC and NAc. We also found that both aripiprazole and haloperidol, but not bifeprunox, activated the Dvl-GSK3β-β-catenin signalling pathway in the NAc.

A wide range of evidence has identified reduced phosphorylation levels and elevated GSK3β protein levels in the brains of schizophrenic patients, indicating hyper-activity of GSK3β in schizophrenia [19,20]. In addition, antipsychotics, including aripiprazole and haloperidol, have been shown to be able to induce inhibition of GSK3β function in various brain regions [8,11,12,13,21]. In the present study, both aripiprazole and haloperidol were able to increase the phosphorylation levels of GSK3β (the ratio of p-GSK3β/GSK3β) in the NAc and PFC (only for aripiprazole), which is not completely consistent with the findings in previous studies [7,8,11,12,13,15,21]. It should be noted that the present study used oral administration to deliver the drugs (for one week) to mimic the clinical situations, which is different from the methods of other previous studies (e.g., intraperitoneal and subcutaneous injection); the dosages of antipsychotics used in this study are transferred from recommended clinical dosages, which are lower than those in previous studies [7,11,12,13,15,21]. Therefore, the results of the present study might be of more significance for clinic. However, whether these discrepancies are caused by different drug delivering methods requires further investigations.

However, the effects of aripiprazole and haloperidol on GSK3β were not completely consistent in every brain region in the present study. Therefore, by comparing the effects of aripiprazole with those of haloperidol, we may further understand the mechanisms of aripiprazole and elucidate its unique clinical profile. The present study has demonstrated that aripiprazole, but not haloperidol, increased the phosphorylation levels of GSK3β in the PFC. This effect is consistent with the result of our previous acute study [8] and another chronic *in vivo* study [7]. Since prefrontal dysfunction is linked to the negative symptoms of schizophrenia [22,23], it is suggested that suppression of GSK3β function in the PFC is very likely to contribute to the effects of aripiprazole on the negative symptoms of schizophrenia, which cannot be achieved by haloperidol [7,8]. Moreover, we have observed that aripiprazole increased GSK3β phosphorylation levels in the NAc in the present and previous acute study [8]; haloperidol also showed similar effects in the NAc presently and previously [8,12,21]. It is suggested that dysfunction of the NAc is related to the positive symptoms of schizophrenia [24]. Therefore, our finding further indicates that inhibition of GSK3β function in the NAc may contribute to the effects of antipsychotics on the positive symptoms of schizophrenia.

It is worth noting that Akt did not change in parallel with GSK3β, which is not consistent with previous reports [12,21,25]. This might be explained by following reasons. First, Roh *et al.* [25] have reported that the phosphorylation of Akt induced by antipsychotics were much shorter in duration than those of GSK3β. In the present study, the animals were sacrificed several hours after the last administration. Therefore, the phosphorylation levels of Akt might have already decreased to undetectable levels. This might be the major reason that we only observed the altered p-GSK3β levels, but not *p*-Akt. Second, there are two phosphorylating sites of Akt—Thr308 and Ser473, both could be affected by antipsychotic drug administration [4,21,25,26,27]. The present study has examined the Thr308 site of Akt only, since phospho-Thr308-Akt was involved in the D_2_Rs-mediated Akt-GSK3β signalling [4,27]. However, Akt phosphorylated with either site induces phosphorylation of GSK3β at Ser9 that was examined in the current study. Thereby, it is possible that the elevated p-GSK3β levels in this study might be induced by phospho-Ser473-Akt from other signalling pathway(s), and further investigations are needed to study this issue. Lastly, GSK3β is a multi-targeted regulator. Antipsychotics might affect GSK3β via alternative pathway(s) rather than the D_2_Rs-mediated signalling pathway, such as the Dvl-GSK3β-β-catenin signalling pathway.

We have examined the effects of antipsychotics on the Dvl-GSK3β-β-catenin signalling pathway. It was observed that both aripiprazole and haloperidol administration increased the expression of Dvl-3 and β-catenin in accordance with the enhanced phosphorylation of GSK3β in the NAc, suggesting that antipsychotics is very likely to affect GSK3β activity via Dvl-GSK3β-β-catenin pathway in this study. However, further studies (e.g., pharmacological or genetic intervention) are required to confirm this suggestion. In addition, it has been reported that antipsychotics (e.g., aripiprazole, haloperidol, clozapine, and risperidone) increased the expression of Dvl-3 and/or β-catenin in various brain regions, including the PFC and striatum [10,12,14]. It is worth noting that the studies by Alimohamad *et al.* [12] and Sutton *et al.* [14] have mixed NAc and CPu together, thus preventing identification of the sub-region(s) in which the levels of Dvl-3 and β-catenin were increased by antipsychotic drug administration. This study has separated NAc and CPu, and demonstrated that antipsychotics affect Dvl-GSK3β-β-catenin signalling specifically in the NAc. Taken together, it suggested that activation of Dvl-GSK3β-β-catenin signalling in the NAc is a common route, through which different classes of antipsychotics exert their effects. Lastly, our results do not show any alteration in the expression of Dvl-3 and β-catenin in the PFC, which is inconsistent with the findings of previous studies [10,12,14]. The exact reason remains unclear. This may be because the previous studies [10,12,14] used intramuscular or subcutaneous injection to deliver the drugs, whereas the current study used oral treatment with different dosages to mimic the clinical situation. Therefore, whether the effect of antipsychotics on Dvl-GSK3β-β-catenin signalling is treatment method-dependent requires further validation. Furthermore, one limitation of this study is that the samples have been investigated by only Western blots method, it is also worthy to further validate these findings using other methods such as qPCR and immunohistochemistry.

Previously, Min and colleagues [9] have investigated the interaction between the dopaminergic nervous system and Dvl-GSK3β-β-catenin signalling, and found that only D_2_Rs directly affected β-catenin distribution in the cell nucleus. The present study used aripiprazole, haloperidol and bifeprunox, all of which have strong affinity with D_2_Rs [18,28]. Haloperidol is a potent D_2_R antagonist, whereas aripiprazole and bifeprunox are D_2_R partial agonists. Previous studies have revealed that the intrinsic activity of aripiprazole at D_2_Rs is weaker than that of bifeprunox (intrinsic activity at D_2_Rs: aripiprazole *vs.* bifeprunox *vs.* dopamine = 86.0% *vs.* 95.1% *vs.* 100%) [22,29]. Our results have demonstrated that administration of both aripiprazole and haloperidol, but not bifeprunox, had significant effects on altering the expression of Dvl-3 and β-catenin in the NAc. Therefore, first, blockade of D_2_Rs is (indirectly) linked to the activation of the Dvl-GSK3β-β-catenin signalling pathway. Second, it is very possible that aripiprazole competes with endogenous dopamine in the normal brain due to its relatively low intrinsic activity to reduce significantly the activity of endogenous dopamine, displaying an overall antagonising effect like haloperidol. In contrast, bifeprunox cannot achieve such effects, probably because of its relatively stronger intrinsic activity at D_2_Rs. Taken together, our study suggests that a relatively low intrinsic activity at D_2_Rs might be essential for a D_2_R partial agonist to achieve meaningful effects via affecting the Dvl-GSK3β-β-catenin signalling pathway.

## 4. Materials and Methods

### 4.1. Animals and Drug Administration

Male Sprague–Dawley rats (aged eight weeks) were obtained from the Animal Resource Centre (Perth, Australia). After arrival, all rats were housed in individual cages under environmentally controlled conditions (temperature 22 °C, light cycle from 07:00 a.m. to 07:00 p.m.), with *ad libitum* access to water and a standard laboratory chow diet. All experimental procedures were approved by the Animal Ethics Committee (Application #AE11/02, 02/2011), University of Wollongong, and complied with the Australian Code of Practice for the Care and Use of Animals for Scientific Purposes (2004). All efforts were made to minimise animal distress and prevent suffering.

Before drug administration commenced, the rats were trained for self-administration of the cookie dough pellets without drugs. After 1-week training, rats were randomly assigned into one of the following four groups (*n* = 6/group): aripiprazole (0.75 mg/kg, t.i.d. (*ter in die*), Otsuka, Tokyo, Japan); bifeprunox (0.8 mg/kg, t.i.d., Otava, Kiev, Ukraine); haloperidol (0.1 mg/kg, t.i.d., Sigma, Castle Hill, Australia); or vehicle for one week. Rats were offered cookies with drugs three times a day (at 06:00 a.m., 02:00 p.m. and 10:00 p.m.) and observed to ensure complete consumption of each pellet. The dosages were translated from recommended clinical dosages based on body surface area according to the FDA guidelines [30,31]. This drug administration method has been well established in our laboratory [32,33]. Specifically, a 0.75 mg/kg aripiprazole, 0.8 mg/kg bifeprunox and 0.1 mg/kg haloperidol dosage in rats is equivalent to ~7.5, ~8, and ~1 mg in humans (60 kg body weight), respectively, all of which are within the used/recommended clinical dosages [34,35,36]. It is worth noting that aripiprazole and bifeprunox induced over 90% D_2_ receptor occupancy in rat brains at these dosages [18], and haloperidol reached approximately 70% occupancy [37], all of which can display physiological and behavioural effects in rodents, without inducing EPS side-effects [18,38,39,40]. After one-week drug administration, all rats were sacrificed between 10:00 a.m. and 12:00 p.m. to minimise possible circadian-induced variation of protein expression. All animals were euthanised by using carbon dioxide. Brains were immediately dissected, frozen in liquid nitrogen and stored at −80 °C until further use.

### 4.2. Micro-Dissection of Brain Samples

Following a standard procedure used in our group [8], discrete brain regions were collected using brain microdissection puncture according to the brain atlas [41]. Briefly, three sections through the forebrain (Bregma 3.30 to 4.20 mm) were collected for the PFC; and three sections through the striatum (Bregma 1.00 to 2.20 mm) were collected for the CPu and NAc, respectively. Tissue dissected was kept at −80 °C.

### 4.3. Western Blots

The Western blot experiments were performed following standard procedures repeated in our previous studies [8,33]. Briefly, frozen tissue was homogenised with 9.8 mL NP-40 cell lysis buffer (Invitrogen, Camarillo, CA, USA) containing 100 μL Protease Inhibitor Cocktail (Sigma-Aldrich, St. Louis, MO, USA), 100 μL β-Glycerophosphate (Invitrogen) and 33.3 μL phenylmethylsulfonylfluoride (Sigma-Aldrich). The homogenised samples were centrifuged, and the supernatants were collected. Protein concentration of each homogenising solution was measured by using the *DC* Protein Assay (Bio-Rad, #500-0111). After denaturing proteins, samples containing 10 μg of protein were loaded into 4%–20% Criterion™ TGX™ Precast Gels (Bio-Rad, Hercules, CA, USA, #5671095) in a Criterion™ Vertical Electrophoresis Cell (Bio-rad, #1656001) at 200 V voltage for 50 min, and then transferred electrophoretically to a polyvinylidene difluoride membrane in a Criterion™ Blotter (Bio-rad, #1704071) at 100 V voltage for 60 min. All membranes were blocked by 5% bovine serum albumin (BSA) for 60 min and incubated in primary antibodies (diluted in 1% BSA) over night. Amersham Hyperfilm ECL (GE Healthcare, Chicago, IL, USA, #28-9068-36) and Luminata Classico Western HRP substrate (Millipore, Billerica, MA, USA, #WBLUC0500) were used to visualise the immunoreactive bands. The immunoreactive signals were quantified using Bio-Rad Quantity One software. The data of each targeted protein were then corrected based on their corresponding actin levels. Experiments were performed in duplicate to ensure consistency.

The antibodies used in the present study to examine the GSK3β-involved pathways were anti-Akt (1:2000; Cell Signalling, Danvers, MA, USA, #4691), anti-phosphor-Akt (Thr308) (1:1000; Cell Signalling, #13038), anti-GSK3β (1:2000; Cell Signalling, #5676), anti-phospho-GSK3β (Ser9) (1:1000; Cell Signalling, #9322), anti-Dvl-3 (1:1000; Santa Cruz Biotechnology, Dallas, TX, USA, #SC-8027) and anti-β-catenin (1:1000; Santa Cruz Biotechnology, #SC-7963). Mouse anti-actin primary polyclonal antibody (1:10000; Millipore, #MAB1501) was used to determine the actin levels. The secondary antibodies were HRP-conjugated anti-rabbit IgG antibody (1:3000; Cell Signalling, #7074) and HRP-conjugated anti-mouse IgG antibody (1:3000; Cell Signalling, #7076).

### 4.4. Statistics

All data was analysed using SPSS Statistics V22.0 program (IBM, New York, NY, USA). Data normality was tested using histograms and a Kolmogorov–Smirnov *Z* test. For statistical evaluation, one-way analysis of variance (ANOVA) was performed if the data was normally distributed. The *post hoc* Dunnett *t* test was then conducted to compare each drug treatment group with the control group. The results of Western blots were normalised by taking the average value of the control group as 100%. The phosphorylation to total signal was calculated using the data from the same blot. Pearson’s correlation test was used to analyse the relationships. A *p*-value of less than 0.05 was considered as statistically significant.

## 5. Conclusions

The present study explored the *in vivo* effects of one-week oral administration of aripiprazole on the GSK3β-dependent signalling pathways in three brain regions that are associated with schizophrenia and the actions of antipsychotics, in comparison with haloperidol and bifeprunox. The current study provides *in vivo* evidence that inhibition of GSK3β activity in the PFC and NAc might be linked to the clinical profile of aripiprazole. This study further suggests that, like haloperidol, aripiprazole can activate Dvl-GSK3β-β-catenin signalling in the NAc, which is probably due to the relatively low intrinsic activity at D_2_Rs.

## Abbreviations

Akt: protein kinase B
CPu: caudate putamen
D_2_R: dopamine D_2_ receptor
Dvl: dishevelled
EPS: extrapyramidal side-effects
GSK3β: glycogen synthase kinase 3β
NAc: nucleus accumbens
PFC: prefrontal cortex
